# Lewinnek zone not “the be-all and end-all” functional planning for acetabular component positioning in total hip arthroplasty

**DOI:** 10.1186/s42836-024-00284-w

**Published:** 2025-01-06

**Authors:** Raffaele Iorio, Edoardo Viglietta, Federico Corsetti, Yuri Gugliotta, Carlo Massafra, Daniele Polverari, Andrea Redler, Nicola Maffulli

**Affiliations:** 1 Sant’Andrea Hospital, Faculty of Medicine and Psychology, Sapienza University of Rome, 00162 Rome, Italy; 2 Sandro Pertini Hospital, Orthopedic and Traumatology Unit, 00162 Rome, Italy; 3 School of Pharmacy and Bioengineering, Keele University Faculty of Medicine, Stoke On Trent, ST4 7QB UK; 4 Centre for Sports and Exercise Medicine, Barts and the London School of Medicine and Dentistry, Mile End Hospital, Queen Mary University of London, London, E1 4DG UK

**Keywords:** Lewinnek, Safe zone, Acetabular positioning, Spino-pelvic, Dislocation

## Abstract

**Background:**

Proper positioning of a total hip arthroplasty (THA) plays a crucial role in the success and long-term survivorship of the implant. Cup positioning within the Lewinnek Safe Zone (LSZ) does not, however, avoid implant dislocation. Thus, the concept of a functional cup position has been introduced. The purpose of this study was to assess the discrepancy between LSZ and the acetabular cup position suggested by the patient’s specific functional planning. The hypothesis was that a mismatch does exist.

**Methods:**

One hundred consecutive patients with primary hip osteoarthritis undergoing primary THA with a personalized functional preoperative planning and patient-specific cup implantation system were enrolled. Anatomical and spino-pelvic functional parameters were recorded and, for each patient, a “safe cup orientation” was suggested. The suggested functional safe zone was compared to the LSZ.

**Results:**

The mean suggested inclination was 39° ± 3° (range 32°–45°). The mean suggested anteversion was 21° ± 3° (range 12°–28°). The patient’s functional acetabular inclination (AI) corresponded to the LSZ in one of the 100 patients, whereas the acetabular anteversion (AV) was outside the LSZ in 8 of the 100 patients. The mean pelvic tilt while standing and sitting were 0.5° ± 7° (range 21°–45°) and −6° ± 16.7° (range −63°–33°), respectively. The mean pelvic incidence was 52° ± 9.7° (range 33°–83°).

**Conclusion:**

When a functional patient’s specific preoperative planning is performed, the LZS does not correspond to the patient’s functional safe zone in about 8% of patients. The concept of a universal safe zone should be revisited, and a functional personalized safe zone may have to be more widely considered.

## Introduction

Proper positioning of a total hip arthroplasty (THA) implant is crucial to the success and long-term survivorship of implants [1–11]. Acetabular cup orientation is considered the most relevant factor [12], and the Lewinnek safe zone (LSZ: inclination 40° ± 10° and anteversion 15° ± 10°) [7] has been traditionally advocated for proper acetabular positioning. However, 60% of dislocations occur even though the acetabular cup is within the LSZ [13–15]. Thus, the concept of a functional cup position, which considers changes in anteversion and inclination according to the postural position of the pelvis, has been introduced [16]. Sagittal postural balance varies with patients, and multiple lumbar and spino-pelvic conditions may affect it [17].

An acetabular cup placed within the LSZ intraoperatively may become unsafe during daily activities, and dislocations may therefore occur. Several authors suggested preoperative assessment of spinopelvic balance through dynamic standing and sitting radiographs, lumbosacral radiographs, or dynamic simulation of the spinopelvic movements [18–21]. The Optimise Positioning System (OPS™, Corin Ltd., Cirencester, UK) platform allows for 3D functional analysis of patients’ hip-spine anatomy and mobility with patient-specific implant positioning. The functional analysis consists of a low-dose CT scan and three lateral radiographs in three functional positions: standing, step-up, and flexed-seated. Hip-spine relationship is assessed through a functional computed analysis and the resultant personalized preoperative planning is provided to obtain the “best” implant orientation for stability and impingement-free ROM.

The purpose of this study was to assess the discrepancy between LSZ and the acetabular cup position suggested by OPS patient’s specific functional analysis. The hypothesis was that a mismatch does exist.

## Materials and methods

**Fig. 1:**
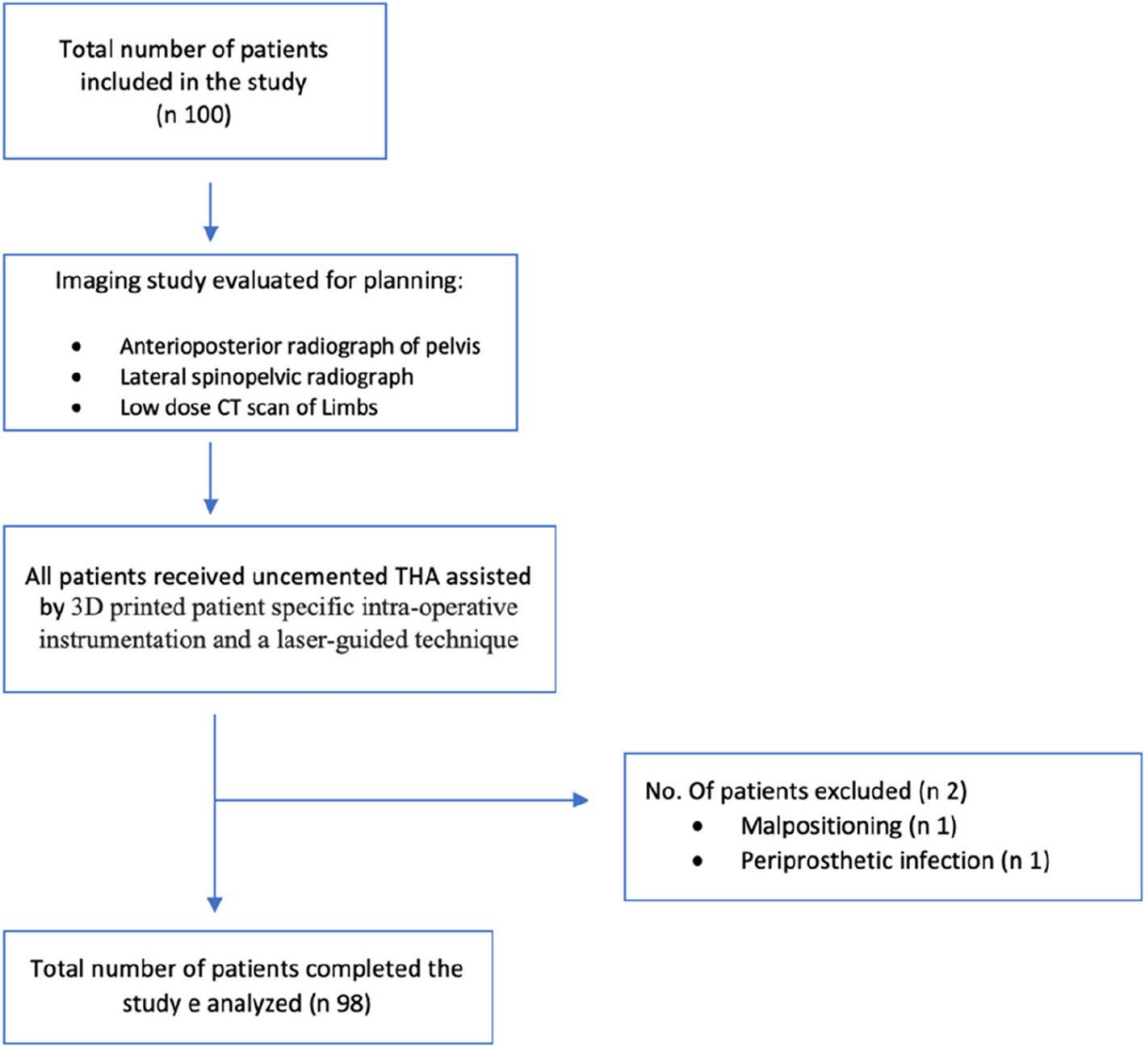
The flow diagram illustrates patient selection and treatment

This was an IRB-approved, prospective cohort, observational imaging study. Between October 2020 and March 2021, 100 consecutive patients with primary hip osteoarthritis undergoing primary THA with a personalized dynamic preoperative planning and patient-specific cup implantation system were enrolled in the study (Fig. 1). All procedures were performed by a single fully trained orthopedic hip surgeon.

Exclusion criteria included: (1) history of previous acetabular/pelvis fracture or hip infections altering the normal bony anatomy, (2) neurological or motor disorders, (3) developmental dysplasia of the hip, and (4) previous spinal surgery.

The imaging study (OPS™, Corin Ltd., Cirencester, UK) consisted of an anteroposterior radiograph of the pelvis, three functional lateral spinopelvic radiographs (standing, flexed-seated and stepping-up), and a low-dose CT scan (mean dose 2.8 to 4.1 mSv per scan) of the lower limbs.

The images were then sent to the manufacturer for analysis.

**Fig. 2:**
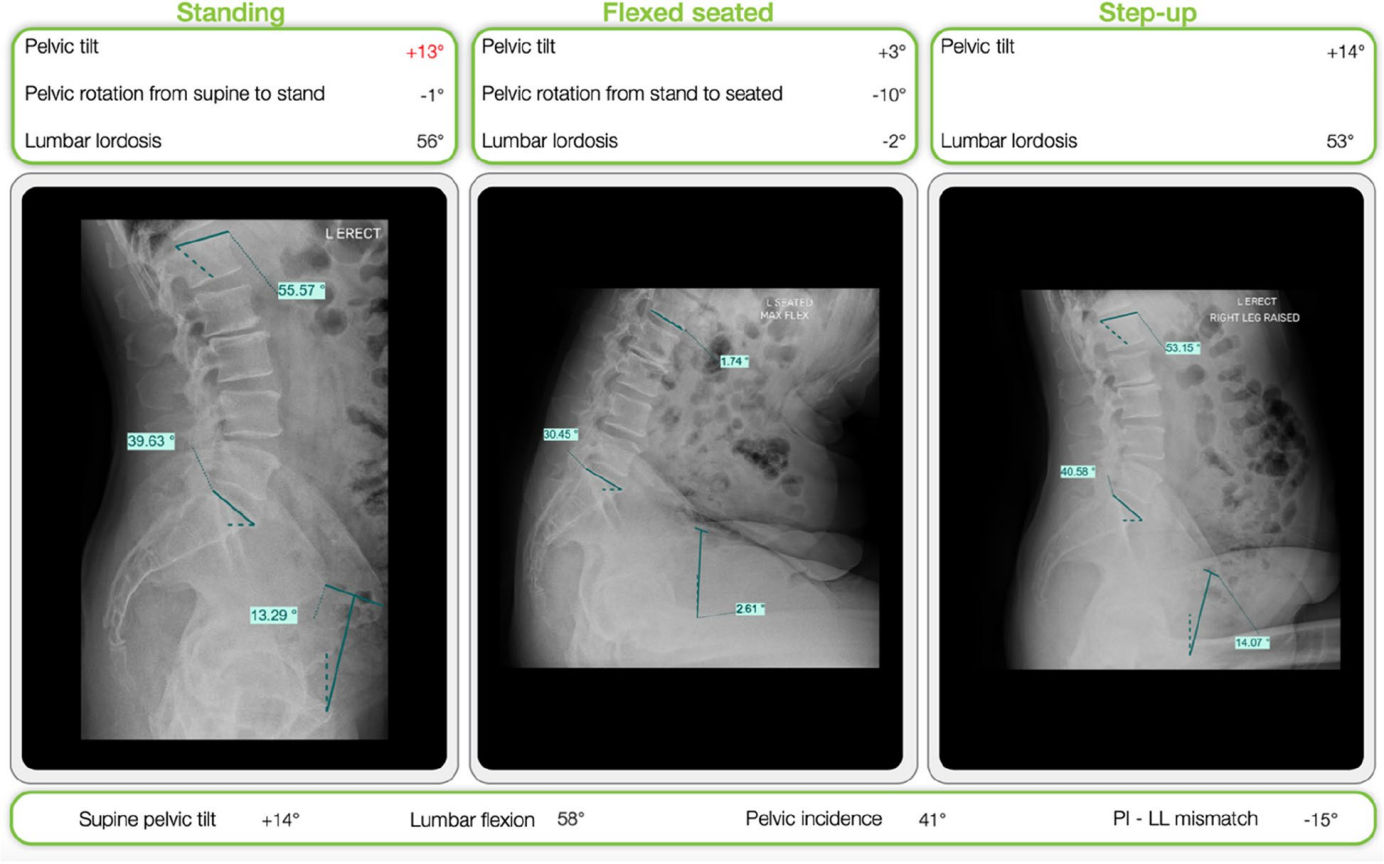
Lateral radiographs evaluated on OPSinsight with pelvic parameters shown

**Fig. 3:**
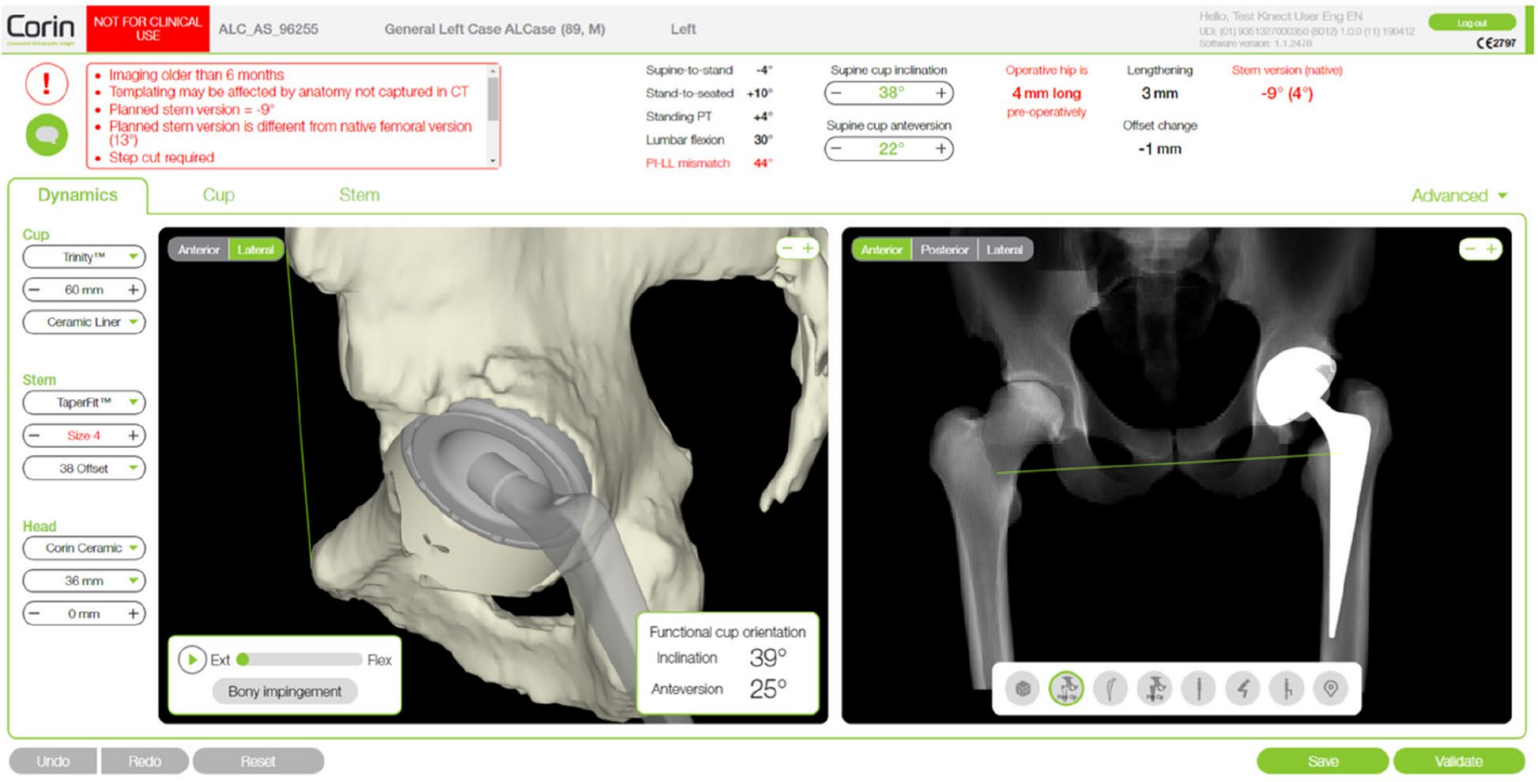
The OPSInsight general layout provides a range of biomechanical and spinopelvic measurements. The implants used in the plan are presented on the left-hand side of the screen. Here, the cup model, cup size, liner material, stem model, stem size, stem neck, head material, head size, and head offset can be viewed and changed

The anterior and lateral radiographs allowed for the evaluation of the following parameters (Figs. 2 and 3).

- Pelvic Incidence (PI): the angle between a vertical line through the femoral head and a line from the mid-sacral plateau and the femoral head which denotes the pelvic orientation in space.
- Sacral Slope (SS): The angle between the sacral plate and the horizontal line. It is reported for the patient’s standing, seated, and step-up positions.
- Pelvic Tilt (PT): the angle between the segment linking the middle part of the sacral plate with the bicoxofemoral axis and a vertical line crossing the interfemoral axis. PT is positive when the hip is in front of the sacral plate and negative when it is behind it.
- Lumbar lordosis (LL) is measured as the angle between the upper-end plate of S1 and the upper-end plate of L1.
- Lumbar flexion (LF) is reported as the change in lumbar lordosis (LL) between the standing and seated positions.
- PI-LL mismatch: the difference between pelvic incidence (PI) and standing lumbar lordosis (LL).

The differences between hip/spine parameters in different postural positions were calculated in degrees as the variation from the starting position and expressed as ΔPI, ΔPT, ΔSS, etc. From the CT scan the following parameters were evaluated (Fig. 2):

- Femoral head size (FSH): the measurement in millimeters of the diameter of the femoral head
- Acetabular size (AS): Acetabular diameter measured in millimeters.
- Acetabular inclination (AI): the angle between the longitudinal axis and the acetabular axis in the coronal plane.
- Acetabular version (AV): the complementary angle to the angle between the anterior and posterior wedges of the native acetabulum (or the acetabular cup, postoperatively) and a second line connecting it to the ischiatic spine.
- Femoral version: the angle between the axis of the native femoral neck and the posterior condylar axis
- Leg length: the sum of the femur and tibia lengths are taken as the distance between the native femoral head and the knee center and the distance between the knee center and the ankle center, respectively. The measurement is performed on the CT scout with the patient in a lying position.

FHS, AS, AI, and AV were measured on the transverse plane running through the middle of the femoral head at its greatest diameter. As far as I am concerned.

A validated algorithm integrated all these parameters to produce a ROM simulation of the normal and prosthetic hip, suggesting an ideal personalized cup position for each patient. The suggested planning included the contact paths simulation presented as polar plots, a proposed cup type, size, and orientation, and, as far as the femoral component is concerned, the level of the osteotomy, the stem type, position, and size, and the estimated change in the leg length and offset (Figs. 2 and 3). According to his own experience and 3D simulations, the surgeon can adjust all these parameters to create the final plan for the operating theatre. All the patients received an uncemented THA through a direct lateral or direct anterior approach at the discretion of the operating surgeon. As described in our initial validation study for the OPS system, femoral osteotomy and acetabular reaming were performed through PSI guides [21]. 3D printed patient-specific intra-operative instrumentation (provided by Corin Ltd., Cirencester, UK) and a laser-guided technique assisted the senior surgeon (R.I) in placing the prosthetic component in the planned position. All patients received an uncemented acetabular cup (TrinityTM cup, Corin Ltd., Cirencester, UK) and a taper-wedged blade stem (TriFit TSTM, Corin Ltd.).

For each patient, the reported data were collected, and the suggested functional patient-specific safe zone was compared to the LSZ. Descriptive and inferential statistics were performed for demographics and patient-reported outcomes. Continuous variables were summarized using means and standard deviations (SD). A two-sample Student *t*-test or Wilcoxon rank sum test was used to identify differences.

## Results

One-hundred consecutive patients were included in the study (53 [53%] and 47 females [47%]). The mean age was 74 ± 12 (range 59–81) years. The mean BMI was 24.3 (range 23–30). The demographic data are reported in Table 1.

**Table 1:** Functional preoperative data of patients included in the study

	**Mean ± SD**	**Range**
Inclination	39° ± 3°	32°–45°
Anteversion	21° ± 3°	12°–28°
Pelvic tilt in standing	0.5° ± 7°	21°–45°
Pelvic tilt in sitting	−6° ± 16.7°	−63°–33°
Pelvic incidence	52° ± 9.7°	33°–83°
Lumbar lordosis while standing	58° ± 11.2°	24°–83°
Lumbar lordosis while sitting	10° ± 12.9°	−14°–49°
Lumbar flexion	47° ± 13.8°	10°–76°
Pi-ll mismatch	−6° ± 9.3°	−24°–19°

The mean suggested inclination was 39° ± 3° (range 32°–45°). The mean suggested anteversion was 21° ± 3° (range 12°–28°). Regarding AI, the LSZ corresponded to the patient’s specific functional cup position in all patients (100% correspondence). Regarding AV, in 8 patients the functional safe zone was outside the LSZ (92% correspondence).

The mean pelvic tilt while standing and sitting were 0.5° ± 7° (range 21°–45°) and − 6° ± 16.7° (range −63°–33°), respectively. The mean pelvic incidence was 52° ± 9.7° (range 33°–83°). The mean lumbar lordosis while standing and sitting were 58° ± 11.2° (range 24°–83°) and 10° ± 12.9° (range −14°–49°), respectively. The mean lumbar flexion was 47° ± 13.8° (range 10°–76°). The mean pelvic incidence-lumbar lordosis mismatch (PI-LL mismatch) was −6° ± 9.3° (range −24°–19°).

The highest degree of suggested anteversion (28°) occurred in a patient with 11° of posterior pelvic tilt from standing to sitting and a subsequent increased risk of posterior dislocation which, however, did not occur. The lowest degree of suggested anteversion (12°) took place in a patient with a flat back (19° of PI-LL mismatch) and subsequent increased risk of posterior impingement and anterior dislocation. Two complications developed.

One patient suffered an anterior dislocation 4 months after surgery. According to the postoperative CT scan, it might depend on an excessive AV (38° vs planned 24°), a likely result of the acetabular guide malpositioning intraoperatively. The patient underwent THA revision and was then removed from the study. One patient suffered from a periprosthetic joint infection (PJI) and was subsequently excluded. Overall dynamic preoperative data are reported in Fig. 1.

## Discussion

The main finding of the present study was that, in 8% of patients, the suggested patient-specific functional cup position was outside the LSZ for the AV. The mean values of the patient-specific functional cup position were 39° ± 3° and 21° ± 3° for AI and AV, respectively. The main difference lay in the suggested cup anteversion, as a markedly higher anteversion (21° ± 3°), as compared to LSF, was found. The highest degree of anteversion (28°) was suggested for a patient with only 11° of posterior pelvic tilt from standing to sitting. The low posterior pelvic tilt reduces the ante-inclination of the cup and may cause an increased risk of posterior dislocation (especially with the posterior approach) during sitting activities [19]. Conversely, the lowest degree of anteversion (12°) was suggested for a patient with 19° of PI-LL mismatch. In case of flat back or high PI, patients presented an increased risk of anterior dislocation, and lower anteversion may be adopted. Regarding cup inclination, the mean value was quite similar (39° ± 3°) to LSZ, but SD was lower. Inter-individual variability did not justify “extreme position” or “surgical mistakes” in most of the patients for cup inclination.

The hip-spine relationship should be constantly taken into consideration when positioning THA implants to produce optimal clinical results [16]. Several parameters need to be evaluated, and various preoperative planning methods have been proposed [20–22]. The OPS functional preoperative study (OPS™, Corin Ltd., Cirencester, UK) allows for the concomitant evaluation of several hip and spine parameters using a validated algorithm and patient-specific planning with computer simulation of the implant postoperative result. Surgeons may accept or refuse and modify the planning to plan the “best” implant position and size in terms of stability and ROM. Lastly, 3D-printed patient’s specific intraoperative instrumentation was provided to obtain the proper position of the implant as planned.

Several recent studies evaluated the risk for dislocation according to the different hip/spine parameters, and most concluded that, in high-risk patients, “a more anteverted cup or more constrained implants (e.g., dual mobility cup) should be considered” [20, 23, 24]. However, no study specified how anteverted the cup should be. The OPS system integrates all the mentioned parameters and produces a dynamic simulation of the reciprocal hip-spine movements. Through this functional analysis, the patient’s specific “best” cup position, implant size, height of femoral neck osteotomy, head length, and others (which are not the focus of the current study) for each patient are provided.

To our knowledge, this was the first study to evaluate a large series of patients’ functional preoperative planning to identify the patient’s specific functional cup position, suggesting that a “general safe zone” in hip replacement surgery may have to be contrasted to the patient’s functional safe zone. Furthermore, the system adopted in the current study simultaneously assessed the “femoral side” of the implant, which was often neglected by most of the preoperative imaging evaluation systems.

We have recently reported imaging and clinical results in the same 100 THAs performed using the OPS system [21]. One dislocation occurred 4 months postoperatively. In that patient, a surgical error resulted in higher postoperative anteversion than planned. Furthermore, a postoperative ± 5° difference from the planned values was found in 78% and 81% of patients for inclination and anteversion, respectively, confirming the high accuracy of the OPS system.

The general validity of the LSZ has been recently called into question [7, 13, 15]. The poor accuracy in achieving proper cup orientation during surgery, the true predictive value in preventing THA dislocation, and the functional acetabular mobility during postural changes represent issues of the LSZ [7, 13, 15, 25–28]. However, most of those studies failed to define a “new safe zone”. Furthermore, many of these studies are based on 2D imaging and/or do not consider the overall hip-spine or the femoral parameters contributing to THA stability [29, 7, 30, 31]. Thus, functional sagittal cup orientation for THA stability is clinically relevant [2, 16, 32, 33].

In a radiographic evaluation of 206 THA dislocations (2% dislocation rate), 58% of them occurred within the LSZ [1]. Hence “the ideal cup position for some patients may lie outside the Lewinnek safe zone” [1], and more sophisticated analyses should be performed to identify the proper cup position for each patient.

Similarly, evaluating a large institutional registry of more than 7000 THAs for a minimum of six postoperative months [2], 147 (2.1%) dislocations were identified, with 54% of them being within the LSZ. Comparison between the patients and a matched group of stable THAs revealed no differences in acetabular inclination or anteversion angles between dislocators and non-dislocators in all the evaluated zones (5° to 40° anteversion and 25° to 55° inclination, with ± 5° boundaries between zones). Therefore, a truly “safe zone” on the acetabular position likely does not exist, and additional research with 3D imaging technology (e.g., CT scans and EOS® imaging) is needed to assess the functional position of the cup based on the hip-spine relationship. Many of the recent studies, that criticized the validity of the LSZ, were based on 2D images and did not consider the 3D functional cup orientation in the different positions of the body. Those studies, however, did not suggest how to identify a patient-specific safe zone and only functional personalized preoperative planning may provide such information.

Tezuca et al. [16] and Heckmann et al. [17] investigated the functional cup position with regard to the sagittal hip-spine motion and assessed them through pre- and postoperative lateral standing and sitting spine-pelvis-hip radiographs. In 86% of the cases, the LSZ matched the sagittal functional safe zone, but 14% of the hips that were inside the LSZ were not within their sagittal functional safe zone. Furthermore, 90% of patients with late dislocation presented an abnormal sagittal hip motion. The main preoperative predictor of being outside the functional safe zone was preoperative femoral motion, followed by a lesser pelvic motion and abnormal pelvic incidence values. Only dynamic studies of the hip may allow for the identification of patients at risk, but it was not possible to clearly define the angles within which the cup should be positioned for a corresponding value of the hip-spine parameters. This, and the lack of an association between the risk of dislocation and cup orientation angles, represent the two main limitations of their study.

In this regard, many studies [1, 16, 20, 23, 24, 26, 32, 34–40] suggested increasing anteversion and/or ante-inclination of the cup in patients with a progressive degenerative spine with coexisting muscle atrophy, or previous spinal surgery. In such a situation, reduced pelvic mobility (i.e., reduced pelvic tilt from standing to sitting and subsequent lesser increase of ante-inclination in the sitting position) makes them prone to posterior dislocation. However, none of these studies clarified the amount of anteversion needed to compensate for spinal stiffness.

The main limitation of the current study consisted of the lack of a correlation between dislocation and coronal/sagittal cup positions. However, the present study aimed to describe the discrepancy between LSZ and the patient’s specific safe zone and was not a study regarding dislocations following THA. Furthermore, we were not able to provide specific cut-off values for the several hip/spine parameters corresponding to specific cup position angles. These could be topics for future investigations. A further limitation of the study was the number of patients included. However, this was dictated by our workload and by the fact that the investigation was undertaken in a national health service hospital, where only limited funding for the use of the OPS system was available.

## Conclusion

In conclusion, the LSZ, which is based on a static coronal position of the acetabulum, is not applicable to all patients, given the recent understanding of the relationship between the lower lumbar spine, the pelvis, and the hip on the sagittal plane. Acetabular inclination and anteversion alter in the different body positions during daily life activities, and these changes are patient-specific. The current study showed that, when a patient’s specific preoperative planning, based on functional sagittal radiographs of the hip and spine in combination with a lower limbs CT, was performed, the LZS did not correspond to the patient’s functional safe zone in about 8% of patients. A greater discrepancy was found for cup anteversion than cup inclination. According to such findings, the concept of a universal safe zone may have to be abandoned and a functional personalized safe zone may have to be considered. Further research is needed to confirm such findings, to assess the relationship between suggested cup position and hip/spine parameters, and to assess the clinical results of the proposed system. Such investigations will require large cohorts, be randomized, involve several surgeons, be multicentered, and be performed over several years.

## Data Availability

The datasets used during this study are available from the corresponding authors on reasonable request.
